# Pre-stroke surgery is not beneficial to normotensive rats undergoing sixty minutes of transient focal cerebral ischemia

**DOI:** 10.1371/journal.pone.0209370

**Published:** 2018-12-28

**Authors:** Michaela Bayliss, Melissa Trotman-Lucas, Justyna Janus, Michael E. Kelly, Claire L. Gibson

**Affiliations:** 1 Department of Neuroscience, Psychology & Behaviour, University of Leicester, Leicester, United Kingdom; 2 Preclinical Imaging Facility, Core Biotechnology Services, University of Leicester, Leicester, United Kingdom; Massachusetts General Hospital/Harvard Medical School, UNITED STATES

## Abstract

Experimental stroke in rodents, via middle cerebral artery occlusion (MCAO), can be associated with a negative impact on wellbeing and mortality. In hypertensive rodents, pre-stroke craniotomy increased survival and decreased body weight loss post-MCAO. Here we determined the effect, in normotensive Sprague-Dawley rats following 60 minutes MCAO, with or without pre-surgical craniotomy, on post-stroke outcomes in terms of weight loss, neurological deficit, lesion volume and functional outcomes. There was no effect of pre-stroke craniotomy on indicators of wellbeing including survival rate (P = 0.32), body weight loss (P = 0.42) and neurological deficit (P = 0.75). We also assessed common outcome measures following experimental stroke and found no effect of pre-stroke craniotomy on lesion volume as measured by T2-weighted MRI (P = 0.846), or functional performance up to 28 days post-MCAO (staircase test, P = 0.32; adhesive sticker test, P = 0.49; cylinder test, P = 0.38). Thus, pre-stroke craniotomy did not improve animal welfare in terms of body weight loss and neurological deficit. However, it is important, given that a number of drug delivery studies utilise the craniotomy procedure, to note that there was no effect on lesion volume or functional outcome following experimental stroke.

## Introduction

Cerebral ischemic stroke is a leading cause of death and adult disability. However, current available treatments are limited in both their utility and effectiveness. In fact, tissue plasminogen activator (tPA) is the only specific pharmacological approach with proven efficacy for ischemic stroke [1,2]. However, due to a narrow therapeutic window (<4.5h) and not all stroke patients being eligible for tPA treatment, approximately only 15% stroke patients receive tPA and recanalization rates can be less than 50% [3,4]. The development of safe and effective treatments is a substantial challenge to stroke research. Translation of therapeutic strategies for stroke treatment is dependent on the reproducibility and reliability of animal stroke models. Experimental models of stroke have been developed to understand the pathophysiological mechanisms underlying ischemic stroke and to study neuroprotective and/or neuroreparative strategies.

Although experimental stroke models are demonstrated to have high construct validity [5], they do have significant impact on animal wellbeing including weight loss, risk of mortality and behavioural deficits. Mortality rates vary widely according to species, type and duration of ischemia induced, experimenter experience and post-surgery care and a relatively recent review reports mortality rates anywhere between 0–33% [6]. Problems with mortality are increased as the duration of occlusion is increased and have been shown, in human stroke patients, to occur as a consequence of brain swelling and increased intracranial pressure [7,8]. The majority of rodent ischemic stroke models target the middle cerebral artery with either transient or permanent occlusion. In terms of a closed skull approach this largely involves mechanical occlusion of the middle cerebral artery intra-luminally using a filament along with clips and/or sutures to tie off the artery or applying compression to stop the flow through the artery. As reported by Percie du Sert et al., [9] stroke patients and experimental stroke studies are similar in that they often show a large heterogeneity in outcome measures including morbidity and mortality within a group of animals exposed to a standard ischemic stroke.

A recent study [10] demonstrated, in hypertensive male rats, that a brief pre-stroke surgical procedure (i.e. small cranial burr hole) significantly improved survival without impacting upon the amount of damage or functional deficit produced. However, over 90% of experimental stroke studies are conducted in ‘normal’ animals–thus, it is important to investigate whether this surgical intervention has the same benefit on survival and animal welfare for normotensive rodents undergoing middle cerebral artery occlusion (MCAO). In addition, a number of studies utilise intracranial delivery of drugs (via a small cranial burr hole) and therefore it is relevant to consider what impact that approach may have. Here we report, that in normotensive male rats, pre-stroke surgical procedure did not improve animal welfare in terms of body weight loss and neurological deficit following 60 minutes of MCAO. In addition, such a procedure did not affect the primary outcome measure, i.e. lesion volume, used in the majority of experimental studies or behavioural outcome.

## Materials and methods

### Animals

This study was conducted in accordance with the UK Animals (Scientific Procedures) Act, 1986 (Project License 60/4315) and following institutional ethical approval by the University of Leicester Animal and Welfare Ethical Review Body. All rats were adult male Sprague Dawley (Charles River, Oxford, UK) aged between 11 and 12 weeks at the time of MCAO surgery. Animals were housed in groups of 3 and half of the cages were randomly selected to undergo craniotomy surgery prior to MCAO. A total of 18 male rats were used in the current study: 3 were humanely killed, using an overdose of anaesthetic (pentobarbital) followed by severance of the femoral artery, within the first 48h following MCAO, 1 animal died during MCAO and 1 died at day 26. All experiments are reported in accordance with the Animal research reporting of *in vivo* experiments (ARRIVE) guidelines [11]. On day 28 post-MACO all animals were humanely killed using an overdose of an anaesthetic (pentobarbital) followed by severance of the femoral artery.

### Pre-stroke craniotomy

Six days prior to MCAO surgery the craniotomy group underwent craniotomy surgery. Anaesthesia was induced with isoflurane (induction 5%; maintenance 2% in 100% oxygen). The animal was placed into a stereotaxic frame (Kopf Instruments, Tujunga, CA, USA) and an incision was made down the midline of the scalp to expose the skull. The skull was cleared of all overlying tissue and a 0.9mm burrhole was made -1.6mm lateral to bregma. A 25G needle was used to pierce the dura and the hole was sealed with dental cement. The wound was sealed with subcutaneous sutures and the animals allowed to recover until subsequent MCAO surgery.

### Focal cerebral ischemia

Anaesthesia was induced with isoflurane (induction 5%; maintenance 2% in N_2_O/O_2_, 30%/70%) and body temperature was monitored with a rectal probe and maintained using a heat mat (Harvard Apparatus, Holliston, MA, USA). Sixty minutes of focal cerebral ischemia was induced by occluding the right middle cerebral artery. A small incision was made on the ventral surface of the neck and the right common carotid artery (CCA) was isolated from the surrounding tissue. A 6–0 silk suture was used to permanently ligate the CCA at the base of the incision, and a microvascular clip (WPA, Hertfordshire, UK) was temporarily placed below the bifurcation of the CCA into the internal carotid artery (ICA) and external carotid artery. A 4–0 nylon filament (Droccol, MA, USA) was inserted into the CCA through a small incision and was held in place by an additional 6–0 suture tie. The vessel clamp was removed and the filament was advanced through the ICA to the origin of the MCA. Laser Doppler flowmetry (Moor Instruments, Axminster, UK) was used to monitor the cerebral blood flow (CBF) to the affected area for 5 minutes prior to and 5 minutes following occlusion. Animals were allowed to recover during the occlusion period before being reanesthetised and the filament withdrawn.

### Analgesia and welfare monitoring

Those rats undergoing craniotomy surgery received subcutaneous buprenorphine (0.002mg/kg, Vetergesic), Marcaine at the area of incision (2mg/kg, 0.25% w/v, AstraZeneca, UK), and 1ml subcutaneous saline prior to surgery. Immediately prior to MCAO, all rats received subcutaneous Buprenorphine (0.003mg/kg, Vetergesic) along with Marcaine (2mg/kg, 0.25% w/v, AstraZeneca, UK) applied directly to the skin surrounding the wound site at the point of wound suture. All rats received saline intraperitoneally (2 ml) prior to surgery and subcutaneously (2 ml) immediately following MCAO to prevent dehydration. All rats were administered Carprieve (10mg/kg, 5% w/v, Norbrook Laboratories Ltd., UK) at 4 hours post-MCAO along with sub-cutaneous saline (2 ml) and all rats received paracetamol for 48 hours following MCAO in jelly for self-administration. 24 hours following MCAO, grimace scoring [12] enabled the assessment of post-operative pain levels post-MCAO in order to inform the decision as to whether further analgesia was required, one animal received an additional dose of Carprieve (10mg/kg). Rats were weighed daily from their first surgery until day 28 post-MCAO as an indicator of their general well-being and body weight changes presented as a percentage change compared to pre-MCAO. For each rat, daily observation score sheets were completed to record the presence of any clinical signs. All rats received subcutaneous (2 ml) pre-warmed saline twice daily following MCAO for 48 hours and had unrestricted access to wet mashed pellets, diet gel (76A Purified soft diet, ClearH2O.com), hydrogel (98% sterile water, ClearH2O.com), dry food pellets and water. At days 1, 2, 9, 16, 23 and 28 post-MCAO rats were assessed using a 28-point neurological deficit score which evaluates circling movements, grasping ability and whisker response.

### MRI and image processing

As described previously in detail [13] MRI scanning was performed on a 9.4T Agilent scanner (Agilent Technologies, Santa Clare, CA, USA) with a 310 mm bore diameter and 12 cm inner-diameter gradient (600 mT/m maximum gradient strength), interfaced with a DirectDrive Console. Diffusion tensor imaging (DTI) and T2-weighted (T2w) images were acquired at 48h post-MCAO. Infarct volumes were delineated on T2-weighted images. Apparent diffusion coefficient (ADC) and fractional anisotropy (FA) maps were generated from DTI data. Rats were fixed in a rat holder with a bite bar and ear inserts (RAPID Biomedical GmBH, Rimpar, Germany). Physiological monitoring was achieved using a custom monitoring and gating system (SA Instruments Inc, Stony Brook, NY, USA). Body temperature was maintained at 37°C using a warm air fan and rectal temperature probe. Respiration was measured using a pneumatic pillow. All scans were performed during the light cycle under anesthesia using 1% - 2% isoflurane in oxygen. Radio frequency transmission and reception was achieved with a 72 mm volume coil and 2-channel surface receive coil specifically for rat brain imaging respectively (RAPID Biomedical GmBH, Rimpar, Germany). The rat brain was positioned at the isocenter of the magnet and located with a fast gradient echo scout scan. Shimming of first and second order shims was performed on a whole-brain voxel using the FASTMAP technique [14] and shim quality was confirmed using point resolved spectroscopy (PRESS) of the water peak. T2-weighted images were acquired using a fast spin echo (FSE) sequence with TR/TE = 3000/40ms, 28x28mm field of view (256 x 256 matrix), 22 x 1mm coronal slices and 2 signal averages (scan duration = 6mins 28secs). DTI were acquired using a FSE sequence with TR/TE = 2380/35 ms, 28x28 mm field of view (128 x 128 matrix), 22 x 1.0 mm coronal slices, 2 signal averages, 14 encoding directions, 10.37 G/cm gradient pulse of 5 ms duration and 26 ms separation, maximum b-value = 1024 s/mm^2^ (scan duration = 13 mins). All images were corrected for intensity inhomogeneities introduced by the 2-channel surface receive coil using the bias field correction method in 3DSlicer, Version 3.6 (http://www.slicer.org [15]) Region-of-interest (ROI) analysis of lesion volume was performed on T2-weighted images in ImageJ, Fiji (www.imagej.net [16]).

Image slices including a lesion component were identified and three repeat measurements of lesion area, ipsilateral hemisphere area and contralateral hemisphere area were manually delineated on each slice by an experienced operator, blinded to experimental group allocation. Area measurements were multiplied by slice thickness to calculate volume. Total lesion volume was corrected for the effects of edema by dividing lesion volume by the ratio of ipsilateral to contralateral hemisphere volume on a slice-by-slice basis.

ADC and FA maps were generated from DTI data using the diffusion analysis method in VnmrJ, Version 4.2 (Agilent Technologies, Santa Clare, CA, USA). Lesion core ROIs were identified on thresholded diffusion-weighted images. Linear registration of diffusion-weighted and T2-weighted images was performed using the General Registration (BRAINS) module in 3DSlicer. Following registration, subtraction of diffusion-weighted lesion core ROIs from T2-weighted total lesion ROIs resulted in an estimated penumbra ROI. These ROIs were applied to ADC and FA maps to calculate mean ADC and FA within the core and penumbra of the ipsilateral hemisphere, and were also translated about the midline to provide corresponding contralateral ROIs.

### Staircase test

The staircase task, developed by Montoya *et al*. [17] was used to assess forelimb function following MCAO. Animals were given a minimum of 90 minutes food deprivation prior to testing and completed 3 days of training, with 2 trials per day, prior to the baseline test. The baseline test was completed 7 days prior to MCAO. Post-MCAO tests were carried out on post-surgical days 6, 13, 20 and 27. For the baseline test and all subsequent post-MCAO tests one sucrose pellet (45 mg) was placed on the top step of the staircase and three pellets on the other six steps. Animals had fifteen minutes in which to retrieve as many pellets as possible. The number of pellets retrieved by each paw was recorded. Animals that failed to retrieve any pellets from the contralateral forelimb at baseline were excluded from analysis.

### Adhesive test

The adhesive task was used to evaluate sensorimotor function of the forelimbs following MCAO. Baseline performance was assessed 11 days prior to MCAO and post-MCAO tests were carried out on days 3, 10, 17 and 24 post-MCAO. Round stickers were attached to the forepaws of each animal. The animals were then placed into a clean, empty cage and the time taken to remove the stickers was recorded. All tests were video-recorded to allow for timing confirmation. On each test day animals completed the task 4 times, with a break between trials. The order in which the stickers were placed on the paws were randomised, each animal received the sticker on the right paw twice and the left paw twice. The time taken to remove the stickers were averaged over the 4 trials of each test and displayed as a percentage of the baseline time.

### Cylinder test

The cylinder test was developed by Schallert *et al*. [18] and was used to determine forelimb asymmetry. Rats were placed into a clear Perspex cylinder 20 cm in diameter and 50 cm in length for 5 minutes. Animals were video recorded in each test and a mirror was positioned at the rear of the cylinder so all paw placements would be captured on the video recording. The video recordings were analysed and the number of rears and which forepaw was placed on the wall of the cylinder first was recorded. The cylinder test was conducted on the same days as the adhesive test, with the order of the two tests being randomised.

### Statistical analysis

All statistical analysis was carried out using GraphPad Prism 7. All data are shown as mean ± standard error of the mean (SEM). Two way analysis of variance (ANOVA) was used to analyse behavioural data with time and intervention as the factors, followed by sidak corrected post-hoc comparisons. A Kruskall-Wallis test was used to analyses non-parametric data where two or more means were being compared (FA and ADC data) and CBF, lesion volume and swelling data were analysed using unpaired, two tailed Students t-test. Mantel-Cox test was used to analyse survival data. Significance was set at *P* < 0.05.

## Results

### Cerebral blood flow

All animals showed a decrease in CBF, relative to pre-ischemic CBF values, following MCAO. There was no correlation between percentage decrease in CBF (38.95 ± 4.21%, n = 14) and lesion volume when corrected for brain swelling (41.43 ± 6.64 mm^3^, n = 14; *r* = -0.037, P = 0.90). Additionally there was no significant difference between the percentage decrease in CBF following induction of ischemia between the control group (33.63 ± 5.63%, n = 7) and the craniotomy group (M = 44.27 ± 5.98%, n = 7; t(12) = 1.30, P = 0.22).

#### Survival

Of the 18 animals, 13 survived until the 28-day post-MCAO end point. Survival analysis showed no significant difference in survival rate between the two groups (P = 0.32). One control animal died during MCAO and was excluded from all data analysis. Two animals (1 control, 1 craniotomy) were humanely killed prior to the 48h post-MCAO MRI scan and were excluded from all data analysis. One craniotomy animal was humanely killed following the 48h post-MCAO MRI scan and was included in the lesion/edema data analysis but not included in the behavioural data analysis. Finally, 1 control animal died on post-MCAO day 26 and was excluded from the neurological score and staircase test analysis.

### Effects of craniotomy on lesion volume and brain swelling

**Fig 1:**
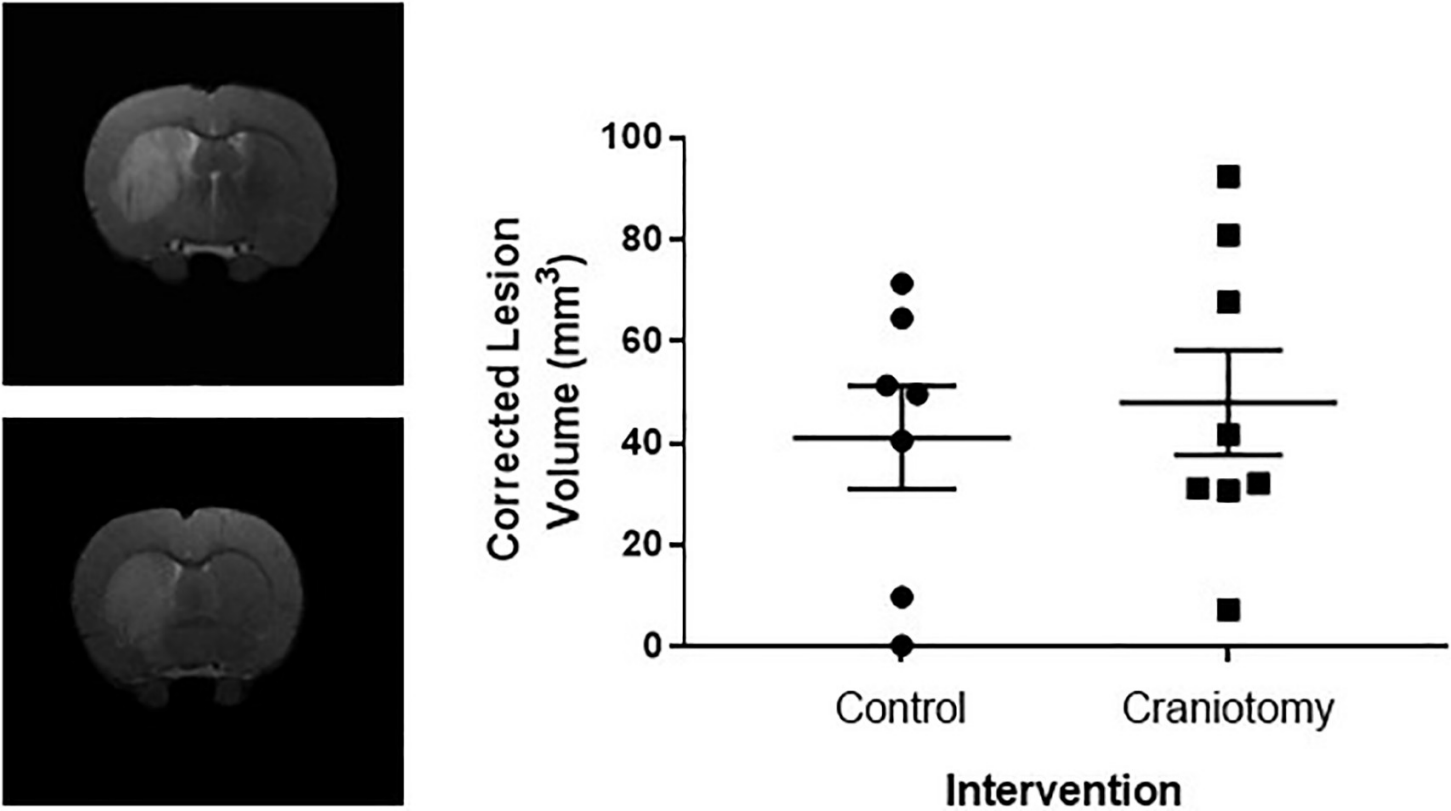
Effect of craniotomy on ischemic lesion volume as calculated from T2-weighted MRI images. (a) Representative T2-weighted MRI coronal images from the control group (a) and craniotomy (b) group of the lesion in the rat brain. (c) Graph showing lesion volume (mm^3^) and corrected against the size of the contralateral hemisphere to account for swelling. There was no significant difference in lesion volume between groups (control n = 7, craniotomy n = 8).

At 48 hours following MCAO there was no significant difference in the amount of ischemic damage present, as indicated by total lesion volume obtained from T2-weighted MRI scans (Fig 1), between the control group (41.15 ± 10.11 mm^3^, n = 7) and the craniotomy intervention group (48.05 ± 10.33 mm^3^, n = 8) when corrected for brain swelling (*t*(13) = 0.475, *P* = 0.846).

In order to test for differences in brain swelling between the control and craniotomy groups, the volume of the ipsilateral and contralateral hemispheres was measured, allowing the volume of the ipsilateral hemisphere to be calculated as a percentage of the contralateral hemisphere. There was no significant difference (t_13_ = 0.49, *P* = 0.63) in the volume percentage of the ipsilateral hemisphere between the control group (102 ± 0.78%, n = 7) and the craniotomy group (102 ± 0.84%, n = 8).

**Fig 2:**
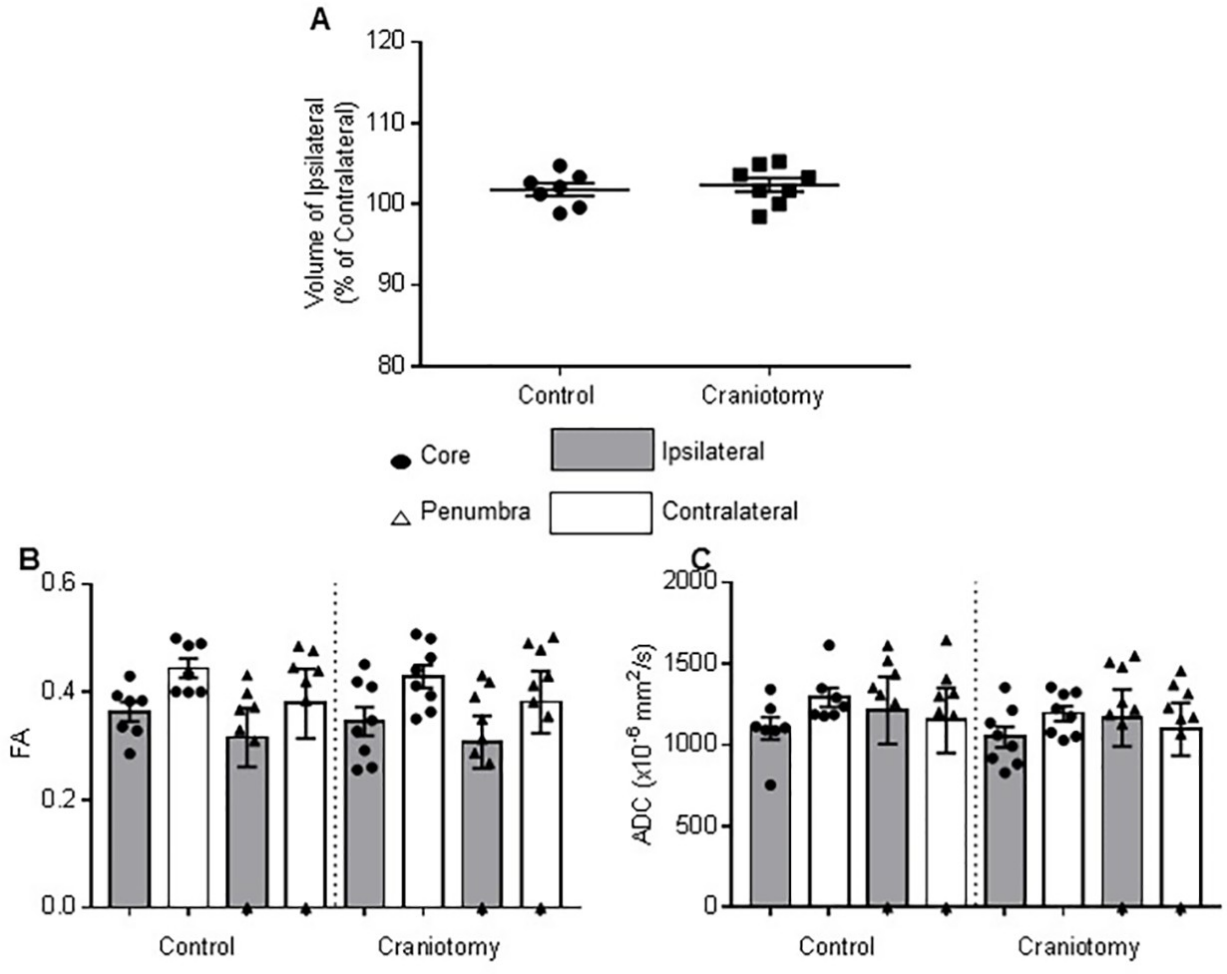
Effect of craniotomy on oedema formation at 48 hours post-MCAO. (a) The volume of the lesioned hemisphere as measured using T2-weighted MRI images, is displayed as a percentage of the unaffected hemisphere. There are no significant differences between the control and craniotomy groups. Craniotomy had no significant effect on the FA (b) and ADC (c) values of either the core or penumbra of the lesion as measured using DTI. Lines indicate mean ± SEM, closed circles are individual data points (control, n = 7; craniotomy, n = 8).

MRI diffusion tensor images were used to measure FA, a measure of local water diffusion anisotropy, and ADC, a measure of the extent of water diffusion, for both core and penumbral ROIs within both the ipsilateral, i.e. ischemic, and mirrored contralateral hemispheres. Within the control and craniotomy groups there was no significant difference between the ipsilateral and contralateral FA in the core (control, P = 0.26; craniotomy, P = 0.45) or penumbral (control, P = 0.82; craniotomy, P = 0.66) regions. There were also no significant differences in FA between the control and craniotomy groups for the core (P = >0.99) and penumbra (P = 0.99) regions of the ipsilateral hemisphere. In addition, ADC values did not differ significantly between the ipsilateral and contralateral hemispheres in the core (control, P = >0.99; craniotomy, P = >0.99) or penumbral (control, P = >0.99; craniotomy, P = >0.99) regions within the control and craniotomy groups. Additionally, ADC values of the core (P = >0.99) and penumbra (P = >0.99) of the ipsilateral hemisphere did not differ between the control and craniotomy groups (Fig 2).

### Indicators of wellbeing following MCAO

**Fig 3:**
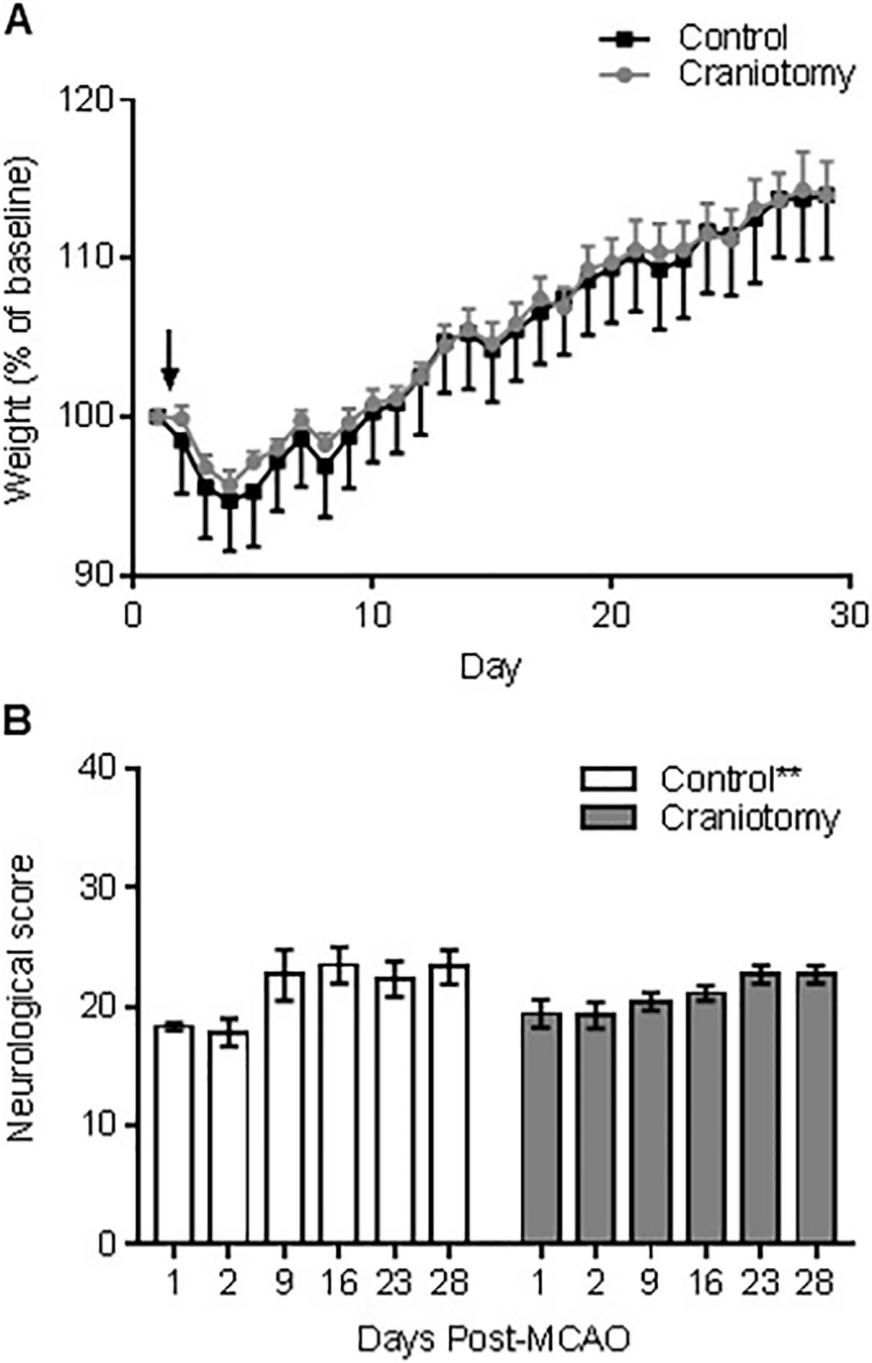
Effect of craniotomy on body weight and neurological score post-MCAO. (a) Body weights were recorded daily as a measure of wellbeing and both groups significantly increased weight over time (P = <0.0001). Arrow represents the point of MCAO. (b) A 28-point neurological score was used to measure functional outcome, craniotomy had no significant effect on neurological score, however there was a significant main effect of time (**P = <0.001). Data is displayed as mean ± SEM; control n = 6, craniotomy n = 7.

Each animal was weighed every day from the day of MCAO surgery up until 28 days post-MCAO as an indicator of general wellbeing (Fig 3A). There was a significant main effect, as analysed by a two-way ANOVA, of time on body weight for all animals (*F*_28, 308_ = 89.6, *P* = <0.01) but there was no main effect of surgical intervention on body weight (F_1,11_ = 0.03, *P* = 0.88) and no interaction between intervention and time (F_28, 309_ = 0.2, *P* = >0.99; control n = 6, craniotomy n = 7).

A 28-point neurological score was used to measure the animals’ neurological outcome following MCAO. There was a significant main effect of time on neurological score (*F*_5, 55_ = 9.35, *P* = <0.01) but no significant effect of intervention (*F*_1, 11_ = 0.1, *P* = 0.75) and no interaction (*F*_5, 55_ = 1.68, *P* = 0.15) between the two groups (Fig 3B). On post-MCAO days 1 and 2 control animals had a significantly worse neurological score compared to day 9 (vs. day 1, t_55_ = 3.29, *P* = 0.03; vs. day 2, t_55_ = 3.67, *P* = <0.01), day 16 (vs. day 1, t_55_ = 3.93, *P* = <0.01; vs. day 2, t_55_ = 4.31, *P* = <0.01) or day 28 (vs. day 1, t_55_ = 3.8, *P* = <0.01; vs. day 2, t_55_ = 4.18, *P* = <0.01). Additionally, the neuroscore on day 2 was significantly worse than day 23 post-MCAO (t_55_ = 3.42, *P* = <0.01). Thus, control animals showed a significant improvement in neurological function from the first 48 hours following stroke, whilst there was no significant differences in score across the test days of the craniotomy animals.

### Functional assessment following MCAO

**Fig 4:**
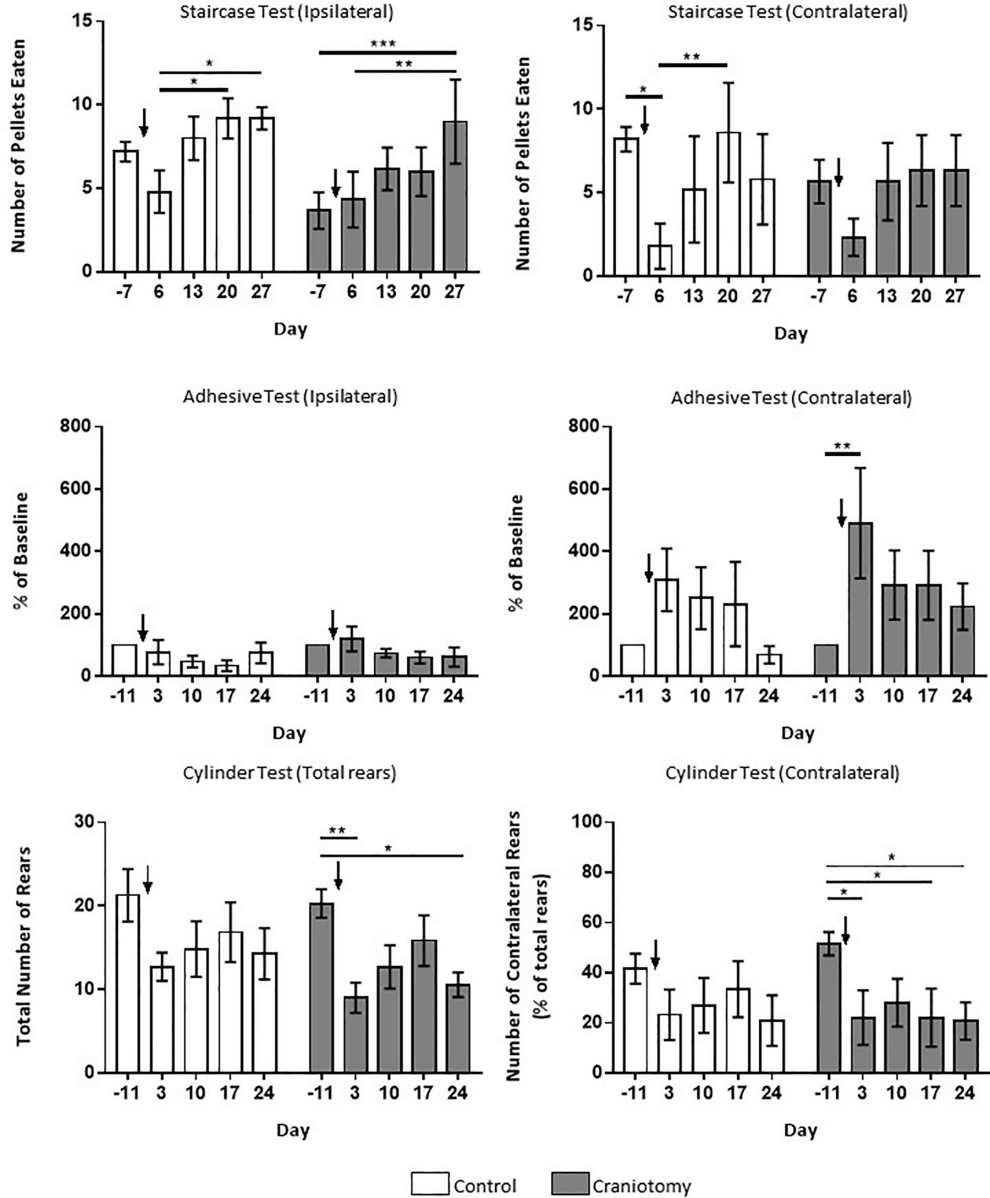
Effect of craniotomy on functional outcome as measured by the staircase test, adhesive test and cylinder test. The time taken to remove the sticker from the ipsilateral and contralateral paw in the adhesive test is displayed as a percentage of the baseline performance, there is no significant difference between the control and craniotomy groups performance (Control, n = 7; craniotomy, n = 7). The staircase test data is displayed as the number of pellets retrieved by the ipsilateral and contralateral paw, there is no significant effect of craniotomy on performance. (Control, n = 5; craniotomy, n = 6). Total number of paw placing rears performed in the cylinder test are displayed and also the percentage of paw placing rears in which the contralateral paw was placed first. (Control, n = 7; craniotomy, n = 7). Arrows represent the point of MCAO. Data is presented at mean ± SEM; * = <0.05, ** = <0.01, *** = <0.005.

The staircase test was used to assess the effect of MCAO on grasping and motor ability of the forelimbs. The number of pellets retrieved by the ipsilateral paw showed a significant main effect of time (*F*_4, 36_ = 8.31, *P* = <0.01) but not intervention (*F*_1, 9_ = 1.10, *P* = 0.32) and there was no interaction (*F*_4, 36_ = 1.50, *P* = 0.22; Fig 4A). *Post hoc* analysis showed that the control group retrieved significantly more pellets on days 20 (9.2 ± 1.2, t_36_ = 3.39, *P* = 0.02) and 27 (9.2 ± 0.66, t_36_ = 3.39, *P* = 0.02) compared to day 6 (4.8 ± 1.28, n = 5). The craniotomy group successfully grasped significantly more pellets on day 27 following MCAO (9 ± 2.52) than on day 7 pre-MCAO (3.67 ± 1.09, t_36_ = 4.50, *P* = <0.01) and day 6 post-MCAO (4.33 ± 1.67, t_36_ = 3.94, *P* = <0.01). This shows that both groups improved their performance and retrieved more pellets over time but neither group performed better than the other (Fig 4A). The number of pellets retrieved by the contralateral (to the site of MCAO) paw was significantly affected by time (*F*_4, 36_ = 5.95, *P* = <0.01) but not intervention (*F*_1, 9_ = 0.07, *P* = 0.80) and there was no interaction (*F*_4, 36_ = 0.84, *P* = 0.84). Results of the *post hoc* analysis showed a significant decrease within the control group between the number of pellets eaten on day 7 pre-MCAO (8.2 ± 0.74) and 6 days post-MCAO (1.8 ± 1.36, t_36_ = 3.52, *P* = 0.01) and the number of pellets retrieved was significantly increased on day 20 (8.6 ± 2.98) compared to day 6 (1.8 ± 1.36, t_36_ = 3.74, *P* = <0.01). No significant differences were found between the time points within the craniotomy group in terms of the contralateral paw, unlike control animals who did have a significant impairment of the affected limb following MCAO, however this impairment was recovered by day 20 (Fig 4B).

Sensorimotor ability following MCAO was assessed using the adhesive sticker test. A two-way ANOVA showed that there was no significant main effect of time on the time taken to remove the stickers from the ipsilateral paw (*F*_4, 48_ = 2.38, *P* = 0.06), no effect of intervention (*F*_1, 12_ = 0.51, *P* = 0.49) and no interaction between time and intervention (*F*_4, 48_ = 0.54, *P* = 0.7; Fig 4C). In terms of the time taken by the contralateral paw to remove the sticker (Fig 4D), there was a significant main effect of time (*F*_4, 48_ = 4.7, *P* = <0.01) but there was no effect of intervention (*F*_1, 12_ = 0.72, *P* = 0.41) and no interaction (*F*_4, 48_ = 0.51, *P* = 0.73). *Post hoc* tests revealed that there was a significant increase in time taken to remove the contralateral sticker by the craniotomy group at day 3 post-MCAO (419.15 ± 176.86%) compared to 11 days prior to MCAO (100 ± 0%, t_48_ = 1.926, *P* = 0.0074), showing a significant impairment of the affected forelimb at this time point.

The cylinder test was used to assess forelimb asymmetry following MCAO. There was a significant main effect of time (*F*_4,48_ = 6.22, P = <0.01) on the total number of paw placing rears made during the cylinder test (Fig 4E), but no effect of intervention (*F*_1,12_ = 0.85, *P* = 0.38) or any interaction (*F*_4,48_ = 0.19, *P* = 0.94). Within the craniotomy group, *post hoc* analysis revealed that there were significantly less rears performed on days 3 (9 ± 1.81, t_48_ = 3.622, *P* = <0.01) and 24 (10.57 ± 1.46, t_48_ = 3.12, *P* = 0.03) compared to baseline at 11 days pre-MCAO (20.29 ± 1.7, n = 7). With regard to those rears performed by the contralateral paw, there was a significant effect of time (*F*_4,48_ = 4.56, P = <0.01) but no effect of craniotomy intervention (*F*_1,12_ = 0.0009, P = 0.98) and no interaction between the two (*F*_4,48_ = 0.63, P = 0.64). *Post hoc* analysis of the craniotomy group’s performance showed that a significantly lower percentage of paw placing rears were performed with the contralateral paw on days 3 (22.14 ± 10.89, t_48_ = 3.08, *P* = 0.03), 17 (22.14 ± 11.6, t_48_ = 3.08, *P* = 0.03) and 24 (20.8 ± 7.52, t_48_ = 3.22, *P* = 0.02) compared to 11 days pre-MCAO (51.63 ± 4.7, n = 7; Fig 4F), suggesting a significant impairment in their contralateral paw, whilst there were no significant differences across the time points in the control groups contralateral limb usage.

## Discussion

In accordance with previous findings [10] this study, in normotensive animals, failed to show any effect of pre-stroke craniotomy on lesion volume and functional outcome up to 28 days following 60 minutes of MCAO. However, despite craniotomy proving beneficial in terms of reducing weight loss and increasing survival post-MCAO in spontaneously hypertensive rats (SHRs) the same benefits were not seen here in Sprague-Dawley rats. A possible reason for this may be that there is only a benefit of craniotomy when there are large lesions as SHRSPs have been shown to suffer significantly larger lesions than normotensive control animals [19–21]. Also, in clinical trials that found reduced mortality following craniectomy in humans, participants were included if they had a National Institutes of Health Stroke Scale of more than 20, which correlates to a moderate to severe stroke [22]. Thus it is possible that this treatment is only beneficial to both animals and humans who have large infarcts. However, as we only investigated the effect following 60 minutes of MCAO we cannot rule the possibility that a longer duration of MCAO might have produced a different effect. In addition, it may be that the size and location of the craniotomy also determines its impact on outcome measures following ischemic stroke.

Transient MCAO by an intraluminal thread is a commonly used model of focal cerebral ischemia in rats (and mice) and, as others have stated [23], it is typically induced for sixty, ninety or one hundred and twenty minutes. As we only tested one duration of MCAO, i.e. sixty minutes, we cannot exclude the possibility that a longer duration may have yielded different results. Various factors affect the extent and severity of the ischemic infarct including occlusion duration, the type of surgical method employed, animal strain and post-ischemia recovery time [24]. Depending on the duration of the occlusion, the method used here typically results in an infarct area encompassing the striatum and the overlying frontoparietal and temporal cortices, as well as a portion of the occipital cortex and some involvement of the thalamus and hypothalamus [25]. Here we chose sixty minutes of ischemia which produced a quantifiable and reproducible infarct area as we were attempting to minimise the impact, from an animal welfare perspective, whilst still demonstrating a quantifiable and reproducible lesion.

Focal ischemia, via MCAO, in SHRs and stroke-prone SHRs tends to result in relatively large infarcts [26, 27] whereas in Sprague-Dawley rats, as used here, MCAO tends to result in smaller infarct volumes which are associated with considerable variability [17, 28]. Despite this variability, Sprague-Dawley rats are the most commonly used species in preclinical stroke studies [29] with approximately 60% of all neuroprotection data coming from the Sprague-Dawley strain [30]. In terms of lesion volume produced the volumes we report here are comparable to other studies which have reported lesions in the range of 150 mm^3^ in Wistar rats and but as small as 30 mm^3^ in Sprague Dawley rats following ninety minutes of MCAO [28, 31]. However, it is difficult to compare lesion volumes between studies due to a number of variables including anesthetic regime, surgical experience, recovery conditions animals are exposed to during recovery and the method and timing used to determine infarct area.

Given the variability seen within our data, although consistent with reports in the literature, and the fact that we saw no differences in our primary outcome measure i.e. lesion volume, it is of course relevant to consider whether our study failed to identify a significant difference between groups due to being underpowered. Although we can use the group means and standard deviations for lesion volume to perform a retrospective power analysis, this tells us that many more animals per group i.e. over 200, would be required to detect a difference in lesion volume, assuming all other experimental parameters were comparable to those described here. Of course, this may suggest the current study is either underpowered or that there is actually no biologically relevant difference between the two groups (i.e. no type II error). The aim of this study was to determine if a pre-stroke craniotomy procedure had any benefit to normotensive animals undergoing stroke studies. The lack of a significant difference in lesion volume between the control and craniotomy groups implies that for the majority of experimental stroke studies, which typically use 8–16 animals per group, and apply similar parameters as described here (type and duration of vessel occlusion), pre-stroke craniotomy will have a negligible effect on lesion volume.

In order to allow animals to progress through the functional tests we used non-invasive MRI scanning for quantification of lesion volume. MRI has been shown, in animal stroke models, to detect the evolving ischemic damage and allows a multi-parametric analysis of tissue status [13, 32]. T2 weighted images allow tissue damage to be characterized which reflects final infarct volume, correlates with clinical deficit and identifies tissue damage early following stroke. T2 weighted images provide an estimate of the vasogenic edema and blood-brain barrier disruption indicative of irreversible damage [33–35]. In addition, we used diffusion-weighted MRI to separate the ischemic lesion into core and penumbra regions and also to quantify regional ADC and FA. The lack of significant difference in core and penumbral volume and for ADC and FA measurements between the control and craniotomy groups further supports the T2-weighted lesion volume data.

Despite the welfare benefits of a pre-MCAO craniotomy procedure in SHRs being identified Ord et al [10] failed to determine the mechanistic actions of the craniotomy that led to reduced mortality and weight loss. Possible mechanisms may include protection from pre-MCAO exposure to isoflurane, although this is unlikely when pre-conditioning happened 6 days prior to MCAO as the protective effect of isoflurane has only been observed when given up to 24 hours prior to MCAO [36]. Furthermore, isoflurane pre-conditioning has been shown to improve neurological reduce infarct volume [37], effects that were not observed in our study or the previous one [10]. It may be that the pre-MCAO intervention reduced the effect of oedema by allowing excess fluid to escape through the gap produced in the dura, therefore preventing a rise in intracranial pressure and improving outcome. However this is not the case in normotensive animals as we failed to show any difference between the craniotomy group and the control group in the amount of swelling in the lesioned hemisphere.

Drug development remains key in the search for effective pharmacological treatments for cerebral ischemic stroke. However, the blood brain barrier (BBB), although critical for maintaining physiological homeostasis within the brain, can hinder the delivery of therapeutic agents to the brain [38, 39]. Although the BBB is disrupted following ischemic stroke this tends to be only partially and at a delayed time point, typically at least 12 hours in human patients, following ischemic stroke [40, 41]. There are many experimental studies which, in the early stages of investigating a potential drug treatment for ischemic stroke, do utilise intracranial delivery of a candidate drug, via craniotomy. Thus, although we did not identify any effect of pre-MCAO craniotomy, on welfare measures, the lack of any effect on lesion volume and behavioural function indicates that the craniotomy method for drug delivery should be expected to have minimal impact on those outcome measures typically used in experimental stroke studies.

## Supporting information

S1 File Original raw data. (XLSX)

S2 File Humane endpoints checklist. (DOCX)
